# A Systems Approach in the Prevention of Undernutrition among Children under Five in Tanzania: Perspectives from Key Stakeholders

**DOI:** 10.3390/nu16111551

**Published:** 2024-05-21

**Authors:** Gasto Frumence, Yannan Jin, Amalberga Kasangala, Saidah Bakar, Gladys Reuben Mahiti, Bertha Ochieng

**Affiliations:** 1 Department of Development Studies, School of Public Health and Social Sciences, Muhimbili University of Health and Allied Sciences, Dar es Salaam P.O. Box 65001, Tanzania; gasto.frumence@muhas.ac.tz (G.F.); gmahiti2011@gmail.com (G.R.M.); 2 Leicester School of Allied Health Sciences, De Montfort University, Leicester LE1 9BH, UK; 3 Department of Preventive Services, Health Promotion Section, Ministry of Health, Dodoma P.O. Box 743, Tanzania; amalberga@yahoo.com; 4 Department of Community Health, School of Public Health and Social Sciences, Muhimbili University of Health and Allied Sciences, Dar es Salaam P.O. Box 65015, Tanzania; saidahmohamed2014@gmail.com; 5 Institute of Health, Health Policy and Social Care, De Montfort University, Leicester LE1 9BH, UK; bertha.ochieng@dmu.ac.uk

**Keywords:** undernutrition, under-fives, culturally sensitive strategies, Tanzania

## Abstract

Undernutrition among under-fives is one of the major public health challenges in Tanzania. However, there are limited studies assessing the contribution of cultural-related strategies in the prevention of child undernutrition in Tanzania. This study aimed at exploring participants’ experiential views regarding developing culturally sensitive strategies for the elimination of child undernutrition for under-fives in Rukwa, Iringa, Ruvuma, Songwe and Njombe regions located in the Southern Highlands in Tanzania. This study applied focus group discussions (FGDs) with forty practitioners to explore culturally-sensitive strategies for effectively preventing child undernutrition in Tanzania. The study participants were purposively selected, and thematic analysis was used to identify themes within the data. This study revealed that district- and lower-level administrative systems should prioritize nutrition interventions in their plans, allocating adequate resources to implement culturally sensitive nutrition interventions, while national-level organs need to strengthen institutional capacity and ensure the availability of funds, skilled human resources and a legal framework for the effective implementation and sustainability of nutrition interventions at the district- and lower-levels. This study highlights that for the successful implementation of culturally sensitive strategies towards the elimination of child undernutrition, there is a need to use a systems approach that allows for collaborative governance whereby different sectors act together to address the persistent malnutrition epidemic.

## 1. Introduction

Globally, childhood undernutrition continues to be a significant issue, especially among children under the age of five. According to the 2023’s estimates from the World Health Organization (WHO) and the United Nations Children’s Fund (UNICEF), approximately 148.1 million children under five experienced stunted growth, 45 million suffered from wasting and 37 million were overweight [1]. Undernutrition among children occurs when there is an insufficient intake of the caloric or energy content of food and nutrients to meet children’s needs to maintain good health [2]. Undernutrition manifests as wasting or low body weight for height (acute malnutrition), stunting or low height for age (chronic malnutrition), underweight or low weight for age and mineral and vitamin deficiencies [3].

Children in Sub-Saharan Africa (SSA) face a significant challenge with stunting, with an average prevalence of 41% [4]. The situation is particularly severe in East African countries, where the prevalence of undernutrition among children under five varies from 21.9% in Kenya to a staggering 53% in Burundi [5]. Reports from Tanzania indicate that generally, 30% of children under five are stunted, and 9% are severely stunted; 3% of children under five are wasted, while 4% are overweight; 12% of children under five are underweight, and 3% are severely underweight. Childhood undernutrition is notably widespread in the southern and southern west highland regions of Tanzania, despite their abundance in food crop production. The prevalence of undernutrition in these regions is higher above the national average of 30%; for instance, Ruvuma has an average of 36%, Songwe (32%), Iringa (57%), Rukwa (50%) and Njombe (50%) [6].

Several studies conducted in both urban and rural areas of Tanzania revealed socio-economic, demographic, children feeding practice, maternal and water hygiene and sanitation characteristics as causes for child undernutrition. Specifically, the causes for undernutrition for under-fives included inappropriate feeding practices such as delaying the onset of breastfeeding and shortening the duration of breastfeeding, inadequate maternal dietary intake before conception or during pregnancy, distance to the water, inadequate literacy and big household family size [7,8,9]. Other reported causes include the following: inadequate knowledge on feeding practices among caregivers/mothers [10] and social–cultural factors such as gender inequality related to dietary practices and masculinity, women’s excessive workload—characterized by multiple domestic roles—cultural taboos prohibiting women and girls from eating certain types of nutrient-dense foods such as eggs and fish and excessive alcohol use among mothers or caregivers [11].

The causes of undernutrition for under-fives in Tanzania align with UNICEF conceptual framework of malnutrition, which indicated three levels of causes of malnutrition. The first level is the basic causes, which is also the focus of our study, including causes such as the availability and control of resources (human, economic and organizational), political factors, cultural factors, environmental factors and social factors. The second level is underlying causes which include insufficient household food, inadequate maternal care/childcare and insufficient health services/unhealthy environment. The third level is immediate causes such as inadequate dietary intake and diseases [12].

Globally, WHO has set ambitious targets to address the childhood malnutrition epidemic. One of these targets aims to reduce the prevalence of stunting among children under the age of five by 40% by the year 2025 [13]. Various stakeholders worldwide are encouraged to invest collective efforts in eliminating malnutrition [14]. Additionally, achieving Goal 2 of the 17 United Nations Sustainable Development Goals (UN-SDGs), which is Zero Hunger, involves ensuring the availability of sufficient food globally. This commitment, agreed upon by UN member states in 2015, reflects their dedication to addressing childhood malnutrition, including undernutrition [15]. According to a recent report, governments, especially those in low- and lower-middle-income (LMIC) groups, have demonstrated a commendable level of commitment to combating poor diets and malnutrition [16,17].

Tanzania, in particular, has made substantial efforts in reducing child stunting and other forms of childhood malnutrition. The country has adopted a comprehensive approach, including the establishment of the High-Level Steering Committee for Nutrition in 2011 and the President’s Task Force on Nutrition and a High-Level Steering Committee for Nutrition in 2023. The Steering Committee comprises Permanent Secretaries from nine key line ministries (the Ministry of Water and Irrigation; Ministry of Education, Science, Technology and Vocational Training; Ministry of Health, Livestock and Fisheries), the Planning Commission, development partners, Civil Society Organizations (CSOs), the private sector and academia. Chaired by the Permanent Secretary in the Prime Minister’s Office (PMO), the committee is responsible for ensuring comprehensive and coordinated actions are taken in response to nutrition challenges in Tanzania [18]. The committee meets biannually to discuss National Multisectoral Nutrition Action Plan (NMNAP) implementation progress and endorse plans set by the sectors responsible for nutrition issues [19].

The involvement of multiple systems such as food, education, health, agriculture and social protection plays an important role in tackling barriers to eliminating malnutrition among under-fives. Examples of barriers identified thus far include inadequate awareness of under-fives’ nutritional issues, poor accountability and functionality and provision of strategic leadership for scaling up nutrition, and inadequate malnutrition and poverty alleviation and reduction in social and economic inequality [20]. A systems approach recognizes that ending malnutrition in all its forms calls for a shared responsibility to tackle the multiple determinants of child malnutrition such as employment status, poverty level, education level, food insecurity, government policies and social economic inequalities [21,22,23]. However, there are limited studies focusing on the identification of various culturally-sensitive strategies in combating persistent undernutrition for under-fives. Therefore, this study aimed at exploring culturally-sensitive strategies for the elimination of child undernutrition in Rukwa, Iringa, Ruvuma, Songwe and Njombe regions located in the Southern Highlands in Tanzania.

### Systems Approach to Prevent All Forms of Malnutrition

It has been advocated that a synergy of systemic interventions is required to prevent all forms of malnutrition because it is a multi-dimensional problem, which has a diversified impact on human growth and wellbeing [24]. Therefore, malnutrition calls for a multi-level and multifactorial response across various organizational systems [25,26]. They are the education system, health system, food system, water and sanitation system and social protection system. All of them can act together to address the persistent malnutrition epidemic. Kruahong et al. [27] argued that a systems approach leads to system-thinking particularly in identifying how different systems can influence the risk factors for childhood malnutrition and enforce integrated strategies to prevent all forms of malnutrition. The systems approach guided the analysis and discussion of the key findings in this paper, because it allowed the researchers to explore various sectors (systems) at different levels (i.e., national-, regional- and lower-level) and assess how various sectoral strategies can work together towards addressing the malnutrition problem among under-fives in Tanzania.

## 2. Methods

### 2.1. Study Design

This study employed a cross-sectional, exploratory–descriptive qualitative (EDQ) research design using focus group discussions (FGDs) to collect the experiential insights of key stakeholders in developing feasible, practical and culturally-sensitive strategies for effectively preventing child undernutrition in Tanzania. An EDQ approach coupled with a thematic analysis of the results is considered the most suitable to meet the purpose of this study and others of a similar kind [28], as it allows researchers to explore and describe the beliefs, perceptions, experiences and practices of stakeholders in the context of developing strategies for preventing childhood undernutrition at local, regional and national scales [28].

We conducted four FGDs to collect data from experts from different national-, regional- and district-level organizations involved in nutritional issues. These organizations include the Ministry of Health, government agencies, research and academic institutions and nutrition coordinators at the regional and district level in five selected regions in Tanzania, believing that, since they come from different sectors at different organizational levels (a systems approach), they may come up with different perspectives regarding feasible and practical strategies for combating malnutrition for under-fives in Tanzania. Therefore, this study aimed at exploring participants’ experiential views regarding developing culturally-sensitive strategies for the elimination of child undernutrition for under-fives in Rukwa, Iringa, Ruvuma, Songwe and Njombe regions located in the Southern Highlands in Tanzania (Figure S1).

### 2.2. Sample Size and Sampling Strategy

Following a formal invitation, 40 experts from the Ministry of Health, government department (Tanzania Food and Nutrition Centre), research (National Institute for Medical Research) and academic (Muhimbili University of Health and Allied Sciences) institutions and nutrition coordinators at national, regional and district levels in five selected regions in Tanzania attended the one-day stakeholder workshop to discuss the development of culturally-sensitive strategies for the elimination of child undernutrition for under-fives in Rukwa, Iringa, Ruvuma, Songwe and Njombe regions located in the Southern Highlands in Tanzania. A purposive sampling technique was used to select study participants who were invited to participate in the workshop. The selection of the study participants was based on the roles they had played in implementing various nutrition-related interventions for eliminating undernutrition among under-fives in the selected regions and districts in Tanzania.

### 2.3. Ethical Consideration

This study was approved by the National Health Research Ethics Review Committee with reference number NIMR/HQ/R.8a/Vol. IX/3610 of 10 February 2021. There was no identified form of harm to the study participants, and all of them signed the consent form before data collection and were reassured that their information would be kept confidential.

### 2.4. Data Collection

Using a similar method of data collection that was described in our earlier paper [11], data collection was conducted in March 2021. The study participants were recruited based on their experience and knowledge in the area of tackling childhood malnutrition at national, regional and district levels. Data collection started with a briefing of the study purpose and objectives to all 40 participants. Informed consent was sought and provided by all participants before they took part in FGDs. Four focus groups were formed with each group comprising 10 participants. A purposive sampling technique was used to select and group FGD participants whereby the research team ensured that each group included at least one medical doctor, a nurse, a health officer, a nutritionist, a pharmacist and an administrator. A FGD guide written in both English and Kiswahili was introduced to participants to facilitate data collection. The guide comprised the general rules and structures to follow during the FGD and open-ended questions to discuss regarding culturally-sensitive strategies for the elimination of child undernutrition in Tanzania. The main discussion questions in all groups were as follows: what are the culturally-sensitive strategies that can be developed for the elimination of child undernutrition in their respective regions and who are the key players in the implementation of the proposed strategies? The FGDs employed a nominal group technique that features a structured group discussion to ensure an interactive session among focus groups’ members on the designated topics. Four Research Assistants (RA) with a minimum of a Bachelor’s degree in Nutrition facilitated the FGDs. In each FGD, one RA moderated the discussions, supported the individual’s contributions to the discussion topics, and helped the group to elaborate, clarify and extend the discussions as appropriate. The second RA took notes of key information including that from non-verbal communication and finally summarized the ideas generated. This data collection method is considered useful for obtaining a diverse range of ideas and perceptions of participants in a structured manner while largely promoting equal input from all members [29]. All FGDs were audio-recorded, and each lasted approximately between 60 and 90 minutes. At the end of the FGD, each focus group had the opportunity to present a summary of their key discussion outcomes and issues raised. This was later followed by an open discussion of the key findings among all workshop participants, which complemented all findings from FGDs. All key issues that emerged during the open discussions were then collected as a part of the workshop key findings.

### 2.5. Data Analysis

Two experienced RAs transcribed verbatim all FGDs’ audio transcripts. This was followed by a translation from Kiswahili to English by the first RA, while the second RA validated the materials by translating them back. Translation and back-translation enabled other team members who are not Swahili speakers to contribute to later data analysis and increased the validity and trustworthiness of the study results [30,31]. Field notes taken by the RAs documented the contextual information, and they were used to triangulate data during analysis. A thematic data analysis approach, applying inductive reasoning, was conducted manually. Three researchers identified and then validated all emerging themes from all translated transcripts, followed by a line-by-line data analysis. Furthermore, an inductive approach was used to ensure that the emerging themes were strongly linked to the data, rather than being influenced by the researchers’ own pre-understanding [25,26]. Three steps were used to analyze data manually: the line-by-line coding of transcripts whereby emerging concepts were identified and developed; an examination and interpretation of codes into descriptive themes; and the condensation of descriptive themes into more abstract analytical themes.

## 3. Results

### 3.1. Socio-Demographic Characteristics of Participants

A total of 40 study participants from Tanzania working in the area of children’s health and malnutrition prevention interventions participated in the FGDs (Table 1). Out of all study participants, 20 (50%) were aged between 35 and 44 years, and 24 (60%) were female. On the other hand, 32 (80%) study participants had a college-/university-level of education. Profession-wise, seventeen (42.5%) were nutritionist officers, four (10%) were medical doctors, five (12.5%) were nurses and six (15%) were health officers (Table 1).

### 3.2. Descriptive Themes Developed from Data Analysis

The analysis of qualitative data generated several sub-themes that illustrated different strategies implementable by various sectors or systems across national, district and community levels and how they can work together towards addressing the undernutrition issues among under-fives in Tanzania as a whole. The proposed strategies are grouped into the community, health facility, district and national levels.

#### 3.2.1. Proposed Community-Level Strategies

At the community level, several sub-themes emerged concerning potential strategies to address child undernutrition. These include involving influential community leaders and people to disseminate nutrition-related messages, creating village by-laws to discourage harmful cultural practices, establishing local economic groups to empower women economically, strengthening the implementation of the village Health and Nutrition Days and making nutrition a standing agenda item in regular Ward Development Committee (WDC) meetings.

##### Involving Influential Community Leaders and People to Disseminate Nutrition-Related Messages

The use of community influential leaders such as faith leaders was also mentioned as a strategy that can lead to dietary behavior changes among community members. It was reported that influential leaders can disseminate nutrition-related messages to people in their respective areas. In addition, the village can also use community health workers (CHWs) to disseminate information on improving caring and feeding practices among pregnant and lactating women. Quoted examples of original statements shared via FGDs are provided below.

*“Use of key influential people in the community to support delivery of nutrition education messages in their respective areas. As we have seen key people can influence the community as it has been seen in different areas. As you know, when influential people speak, their messages are received and respected by the members of the community”*.

(FGD 3, participant 4)

*“Community health workers are very instrumental in delivering messages to both pregnant and lactating mothers with regards to balanced diet they should take for their own health and that of their babies”*.

(FGD 3, participant 6)

In addition, the study participants reported another strategy of engaging mothers-in-law to promote exclusive breastfeeding in order to support the effort towards the elimination of child undernutrition. The study participants noted that mothers-in-law have an influence on breastfeeding practice. For example, if they believed that exclusive breastfeeding (EBF) was not sufficient for babies, they sometimes advise breastfeeding mothers to give their children solid food and water, which may lead to child undernutrition in the long run.

*“On breast feeding let us engage mother in-laws. For example, when I got my second born my mother in-law came. To our surprise she gave the baby some water to drink while we kept saying exclusive. And when we told her the baby is not ready to take water, she replied your baby was thirsty. When I tried to explain she told me all of my children even your wife I treated the same, she used to cry hard and when I give water to drink, she stopped. Therefore, for her it was normal, it is better to communicate well in advance”*.

(FGD 4, participant 5)

The study participants also mentioned that there is a need to have a positive engagement of traditional birth attendants and tradition healers in averting negative cultural practices such as the restriction of women and girls from eating foods that are considered “smelly,” such as fish and eggs, as such practices have adverse or detrimental impacts on both mother and child nutrition and health. The participants perceived that traditional birth attendants and tradition healers have close contact with communities; therefore, equipping them with the right knowledge about nutritional issues and preventative measures will help promote nutritional education across communities and ultimately the effective elimination of child undernutrition. One of the participants said the following:

*“..Some influential people like birth attendants and traditional healers do not have clear and defined knowledge of negative impact regarding cultural practices and taboos on nutrition issues. They need to be sensitized with positive messages and use them to facilitate adoption of positive practices in their communities”*.

(FGD 3, participant 7)

##### Creating Village by-Laws to Discourage Harmful Cultural Practices

The study participants also mentioned that the community leaders should create village by-laws aimed at preventing harmful cultural practices such as excessive alcohol consumption among breastfeeding mothers that drive undernutrition among children in the community and encouraging practices such as the adherence to Water, Sanitation and Hygiene (WASH) practices, eating nutritious foods and strengthening the implementation of the law that allows women to own land, thus empowering them economically. The quote below substantiates this:

*“But also, we think that there should be bylaws in the community preventing gender-based violence; misconceptions; those traditional practices that causes child malnutrition like alcoholism and emphasizing on hygiene, eating nutritious foods and those that favor women to own land”*.

(FGD 2, participant 5)

##### Establishing Local Economic Groups to Empower Women Economically

Regarding empowering women to become independent in making the right decisions on their children’s nutritional feeding, participants suggested that there is a need to establish economic groups to achieve women’s economic empowerment and independency. One of the respondents stated the following:

*“We have also heard about capacitating women economically and this can be done through women groups by taking small loans. Those women groups should be supported so that they become economically independent”*.

(FGD 3, participant 8)

##### Strengthening the Implementation of Village Health and Nutrition Days

The study participants emphasized the need for community leaders to strengthen the village’s Health and Nutrition Days by creating competitions and awarding families that perform well in childcaring and tackling malnutrition issues and establishing radio programs with nutrition messages delivered for dietary behavior change. The village Health and Nutrition Day is a physical community-based platform that provides easier access to integrated nutrition and health services to community members. It aims to bring comprehensive health and nutrition services to the grassroots communities, especially those that would not normally be reached by healthcare professionals. As suggested by the FGD participants, the proposed competitions led by the village are likely to improve childcaring and reduce the prevalence of undernutrition in the community, as one of the respondents had said the following:

*“At the community level, leaders can create competition whereby families that feed balanced diet to their children and do not have children with undernutrition can be shortlisted and compete for certain awards. The best families should be recognized by the village leadership”*.

(FGD 4, participant 9)

##### Making the Nutrition Issue a Standing Agenda in Ward Development Committee Meetings

At the ward level, making nutrition a standing agenda item in WDC meetings was proposed in the FGDs as an important strategy for eliminating child undernutrition at the community level. FGD participants reported that the WDC meets regularly to discuss social and economic development issues at the ward level while closely overseeing any development issues. Therefore, it is important to make raising and discussing about nutritional issues a permanent agenda in their meetings in order to build a culture of making close follow-ups on various nutritional interventions that are being implemented at the village level.

*“When the WDC meets, they should make sure the nutritional issues become an agenda in every meeting as this will show seriousness among our local leaders in making follow up on these issues at the community level”*.

(FGD 4, participant 9)

Related to that, FGD participants mentioned that if nutritional issues are discussed at the ward level, then ward leaders will provide reports to the Local Government Authorities (LGAs) at the district level regarding the implementation of interventions for tackling nutritional issues at the ward level and make LGAs aware of the progress made regarding various interventions taking place at the community level.

*“You know, through regular reporting to the district level, ward level becomes an important linking organ with the district authorities on this matter”*.

(FGD 4, participant 7)

#### 3.2.2. Proposed Health Facility-Level Strategies

Two sub-themes were generated with regards to strategies to eliminate child undernutrition at the health- facility-level. These include the following: (1) the continuous provision of education on breastfeeding to pregnant and lactating women and (2) developing ready-to-use therapeutic foods (RUTFs) based on locally available food ingredients.

##### Continuous Provision of Education on Breastfeeding to Women

The study participants mentioned that at the health-facility-level, healthcare workers should provide education regarding exclusive breastfeeding for infants to pregnant and lactating women on a regular basis. According to the FGD participants, the provision of breastfeeding education to women should be a permanent agenda as part of health education, promotion and support to the community members until full compliance to the breastfeeding recommendation is achieved.

*“A provision of breastfeeding education and support to women should not be on ad hoc basis but a permanent agenda by the healthcare workers in all health facilities”*.

(FGD 3, participant 4)

Related to the provision of continuous education on exclusive breastfeeding to women, FGD participants also emphasized that there is a need to ensure that health service providers and community health workers are being exposed to updated knowledge on nutritional issues to enable them to manage malnutrition using locally available foods, as remarked by one of the FGD participants.

*“My friends, there are new things in this field of nutrition, therefore the government in collaboration with other key stakeholders should impart such new knowledge to health workers, otherwise, they will be outdated and unable to manage the challenge of malnutrition in the areas”*.

(FGD 3, participant 7)

##### Developing Ready-to-Use Therapeutic Food Based on Locally Available Ingredients

Participants stressed that developing RUTFs based on locally available ingredients will be one of the most promising local innovations for dealing with childhood malnutrition in the study regions. Furthermore, healthcare workers at the health-facility-level should support such an innovative idea to help communities use their locally available food resources and receive regular trainings on how to promote and facilitate such an innovation and its implementation.

*“In these regions, people have plenty of local food stuff which if properly processed can be used to eliminate malnutrition for our children”*.

(FGD 2, participant 2)

#### 3.2.3. Proposed District Local Authority-Level Strategies

The analysis of the findings from FGDs also generated a few sub-themes regarding the district local authorities’ role in the elimination of children’s undernutrition. The generated themes include the following: (1) the allocation of a special budget for implementing nutrition-based activities within councils, (2) establishing farming classes (or schools) to train community members on farming diversified foods to improve access to nutritionally diverse diets, (3) strengthening school feeding programs, (4) creating an enabling environment for nutrition professionals to effectively carry out their duties and responsibilities, and (5) initiating campaigns to educate communities on the estimation of food availability throughout the year.

##### The Allocation of a Special Budget for Implementing Nutrition Based Activities within Councils

This was raised in the discussion as an important district-level strategy towards the elimination of undernutrition among children. According to the study participants, they claimed that LGAs are supposed to allocate TZS 1000 (USD 0.38) per under-five child for implementing nutrition-based activities in councils; however, not all councils had been allocated such funds, and even though some councils have been allocated the funds, they did not disburse the full amount for implementing the planned interventions as expected.

*“I know it is not easy but the council authority should allocate and disburse the funds for implementing nutrition interventions as directed by the central government, this will facilitate the implementation of planned nutritional activities in the council, this money if not used can be directed to accomplish other non-nutrition activities at the council”*.

(FGD 3, participant 5)

##### Establishing Farming Classes to Train Individuals on Farming Diversified Foods to Improve Access to Nutritionally Diverse Diets

It was also mentioned that the LGAs have the responsibility of establishing field farming classes (farmer-field-schools) in their councils so as to train community members in growing diversified foods that will eventually improve access to nutritionally diverse diets. Farmer-field-schools are mobile schools that proactively reach all communities and bring experts in nutrition to educate the community members about producing a wider range of foods outside their usual staple food categories to diversify and enrich the nutritional intake of community members, such as teaching them to grow different fruits and vegetables for domestic consumptions.

*“Our poor people are used to produce the common staple-based diets such as maize and beans, they need to be educated about producing diversified dietary foods as a strategy to eliminate malnutrition especially for the children”*.

(FGD 4, participant 1)

Another FGD participant remarked that

*“Such kind of knowledge can be conveyed to the communities through field class commonly known as ‘shamba darasa (farm class)”*.

(FGD 4, participant 4)

##### Strengthening School Feeding Programs

The discussions also pointed out that the district authorities should strengthen school feeding programs in collaboration with the Ministry of Education and make such programs compulsory for all primary schools to take on in order to ensure that children are not starving while studying.

*“Children in our primary schools should not starve, councils should strengthen school feeding program as part of the strategies to eliminate children malnutrition in our communities”*.

(FGD 4, participant 6)

##### Creating an Enabling Environment for Nutrition Professionals to Effectively Carry out Their Duties and Responsibilities

This initiative was mentioned as a district-level strategy to fight against children’s undernutrition. The study participants said that the district health officers need a supporting work environment that provides transport services to facilitate their movement to the communities to provide education on key health-related practices such as exclusive breastfeeding and the production of diversified food. They further raised the concern that currently there is no adequate budget allocated to nutrition officers for implementing their field activities.

*“My advice is that the council need to allocate sufficient budget for nutrition officer to carry out their field activities as per their work plan”*.

(FGD 4, participant 3)

##### Initiating Campaigns to Educate Communities on the Estimation of Food Availability throughout the Year

The district authorities and especially the Department of Agriculture were also seen as responsible for initiating and supporting campaigns to educate communities on food production and methods of food estimation for both domestic use and for sale in order to ensure an adequate availability of food throughout the year. As reported by the FGD participants, some of the households cannot keep enough food in their stock because of a lack of knowledge to estimate how much food is required for the family throughout the year. Therefore, the study participants recommended that the district authorities need to support nutrition officers to provide education to households regarding the estimation of food for their domestic use and for sales.

*“The district councils must support the nutrition officers to attend village assemblies to educate the communities about food estimate as this will ensure households have adequate food throughout the year”*.

(FGD 4, participant 6)

It was also emphasized that the district authorities need to monitor the nutrition services and activities delivered at grassroots (community) levels as per the contract signed between the President’s Office, Regional Administration and Local Government (PO-RALG) and LGAs. The Village Executive Officers are tasked under the supervision of LGAs to monitor the implementation of nutrition activities by CHWs at the village level. The following quote further elaborates this.

*“It is true there is contract with the community that is one thing. But also, we have indicators included in the reports that are not captured and there is nobody to follow them up, there is no monitoring”*.

(FGD 2, participant 4)

#### 3.2.4. Proposed National-Level Strategies

The findings from FGDs generated four sub-themes regarding national-level strategies for the elimination of children’s undernutrition. They include the following: (1) mainstreaming nutrition into primary school education curricula, (2) creating national forums for discussing nutritional issues, (3) coordinating multisectoral nutritional activities, and (4) conducting regular supervisions of the implementation of nutritional interventions at lower levels.

##### Mainstreaming Nutrition into Primary School Curriculum

During FGDs, the study participants emphasized on the timely need to integrate nutritional knowledge into the primary school education curricula. This intervention is believed to drive the elimination of the existing bad cultural beliefs and harmful dietary practices that have played a big part in accelerating the persisting problem of child undernutrition in the communities. The study participants noted further that if primary school pupils will be imparted with adequate nutrition knowledge, they will become community-change agents when they become adults, as expressed by one of the study participants in the following quote:

*“Our request is for the ministry of education to incorporate nutrition aspects in the existing curricula for the primary schools especially those targeting bad traditions. You know, when they are trained, child undernutrition will be a history in our society”*.

(FGD 3, participant 9)

##### Creating National Forums for Discussing Nutrition Issues

The FGD participants reported that national-level organs including the Prime Minister’s Office, President’s Office responsible for Regional Administration and Local Government, Ministry of Health, government agencies and other stakeholders should create national forums that will draw people with interests and up-to-date insights in nutritional issues across all life stages to discuss solutions and strategies, such as measures to eliminate poor cultural practices that have been leading to child undernutrition in the country.

*“The government needs to establish national forums for discussing nutritional issues, as through it, some bad cultural beliefs hindering elimination of undernutrition for children may be part of the agenda. This is our views”*.

(FGD 2, participant 6)

The study participants also reiterated that the creation of such national forums will provide opportunities for national-level organs to discuss the progress of the compact agreement or performance contract that was signed between the Minister responsible for the Regional Administration and Local Government Authorities (on behalf of the Vice President) and Regional Authorities on scaling up the implementation of nutritional activities at Regional and LGAs. They emphasized that the forum would enable participants to discuss the status of the completion of tasks stipulated in the compact agreement, such as the allocation of resources per child for nutrition, recruitment of nutrition officers and implementation of nutrition-sensitive interventions across sectors.

*“The compact agreement was an important imitative by the government, therefore, we think that an establishment of national forum will help to discuss its implementation progress”*.

(FGD 3, participant 5)

##### Conducting Regular Supervisions of the Implementation of Nutrition Interventions at Lower Levels

The study participants said that the national-level organs including the Ministry of Health, Agriculture and other government agencies should regularly supervise the implementation of different nutritional interventions aimed at improving the nutritional and health status of people at national, regional and district levels. For instance, national officials need to know how the national nutritional guidelines are being implemented at lower levels particularly given the existence of an inadequate number of nutrition personnel at the grassroots’ level. The study participants highlighted that regular supervision in interventions is more important at the lower levels, because the problem of the shortage of nutrition personnel has made community health workers (CHWs) shoulder the responsibilities of providing nutritional services such as health promotion to the communities, despite the fact that they are not very knowledgeable about nutrition as a subject. This information is validated by the quote below from one study participant:

*“We have employed one nutritionist at regional and council but not at community level. We do not have nutritionist at community level who can impart knowledge appropriately. Community health worker is left to deliver nutritional knowledge to the community instead. This community health worker is not employed, is engaged in own activities most of the time, and when it comes to impart nutritional knowledge to the community, it is done partially, therefore supervision is very crucial”*.

(FGD 4, participant 8)

##### Effective Coordination of Multisectoral Nutrition-Focused Activities

The majority of the study participants noted that integrated effective coordination within the nutrition sector and between other sectors is of paramount importance for identifying the underlying multifactorial causes of childhood malnutrition such as social–cultural factors and finding culturally-sensitive solutions to address them.

*“You know, there are many factors contributing to undernutrition to our children that need to be effectively coordinated by many sectors. You see, our people lack safe and clean water, they do not know balanced diet to give their children, …as we discussed here, there are also myth about some of the food staff which are prohibited to pregnancy women. It is not an easy task to coordinate all of these”*.

(FGD 2, participant, 6)

As noted by the study participants, there are many sectors and actors involved in promoting nutritional activities in the country, which imply an existence of different guidelines, strategies, services and resources. This drives the need to integrate all of them into a single system to ensure the united and consistent accountability of all players towards the agenda of eliminating child undernutrition in the Southern Highlands region and in Tanzania as a whole.

## 4. Discussion

This study provided an in-depth understanding of various feasible and culturally- sensitive strategies that can be practically implemented by sectors at different organizational levels (from community- to national-level systems) for addressing the ongoing issue of undernutrition among under-five children in Tanzania. It is worth noting that during data collection and the analysis of the findings, there were no differences in opinions, attitudes or perceptions between focus groups regarding culturally-sensitive strategies for eliminating child undernutrition. In addition, the findings from this study did not find different strategies for eliminating child undernutrition in the Tanzania regions under study. The main reason could be that all regions are not only from the same zone (Southern Highlands) of the country, but they also implement similar interventions regarding the elimination of child undernutrition and that the National Multisectoral Nutrition Action Plan guides the implementation of those interventions.

This study revealed that at the community level, there are different actors who can play key roles in addressing the problem of undernutrition among under-five children. Influential leaders such as faith leaders can be used to change people’s behavior through educating their followers about the importance of the use of nutritional food for their children. The engagement of CHWs and traditional birth attendants and tradition healers may also be used to disseminate information aimed at improving caring and feeding practices and on averting negative cultural practices that have negative bearing on nutrition among women, particularly pregnant and lactating mothers, as well as children. It is well documented that CHWs play a critical role in community-based healthcare, because they are not only close to vulnerable individuals and communities, but they are also close to the healthcare system particularly the community health system [32]. In addition, they provide direct social and healthcare services including nutrition education to families and the community [33]. It was also reported in Bangladesh that a home visit conducted by CHWs had better message deliveries and contact-coverage among children aged 6–59 months to improve the distribution of micronutrient powders in rural Bangladesh [34]. Similarly, in the case of handling severe acute malnutrition (SAM) in countries including Angola, Bangladesh, Ethiopia, India, Malawi, Mali, Pakistan and South Sudan, CHWs demonstrated their advantageous position and capacities in identifying and treating uncomplicated cases of SAM in a cost-effective manner, by achieving cure rates above the minimum standards and reducing default rates to less than 8% [35]. The direct access of CHWs to communities played a pivotal role in the early detection and treatment of SAM, which increased the coverage of SAM in a cost-effective manner. Yet, the benefits of using CHWs come with a few challenges. To enable CHWs to deliver effective coverage, clinical outcomes and qualitative care, prerequisites are essentially required, including providing adequate nutrition training and close supervision of CHWs, creating incentives either in a financial or social recognition form to motivate CHWs, and giving a sufficient supply of resources and commodities such as ready-to-use therapeutic foods to communities [35]. The ultimate underpinning force to drive cohesive and consistent performance among CHWs is good nutrition education and a culturally-sensitive nutrition policy and implementation practice.

Sharing similar notions, the FGD participants pointed out that in order to effectively use CHWs and traditional attendants and healers as change agents for solving nutritional issues, there is a need to give them the right knowledge about nutrition and its related issues. This implies that nutrition officers and other key stakeholders at the district and ward levels have a role to educate CHWs and traditional birth attendants in community-level health systems on the best childcaring and feeding practices. In Brazil, it was reported that offering training to CHWs enhanced their capacity to meet new health challenges which are rapidly emerging. This kind of training should focus on the training needs for CHWs related to the needs of healthcare that they are dealing with in their respective communities [36]. Implementing training needs-based programs for CHWs was also reported in another study as an intervention to help fill gaps in effectively providing health services such as mental healthcare in underserved U.S. Latino communities [37]. The capacity-building program for CHWs is recognized not only as an intervention to advance their capacity, but also to motivate CHWs to improve their performance so that they will feel valued and cared for. Therefore, capacity-building programs for CHWs and other workers working in the community health system must include among other things training to improve their knowledge on nutritional issues so as to support interventions aimed at eliminating malnutrition for under-five children. It is also important to include topics on nutritional issues in the training curricula that offer educational sessions to CHWs and other extension officers.

The empowerment of CHWs and other community leaders on nutritional issues will also support the eradication of poor cultural practices including the influence of mothers-in-law in discouraging mothers from exclusive breastfeeding, because they believed that it was insufficient for their grandchildren. Such a practice has been reported to contribute towards exacerbating child undernutrition. It is important for CHWs and other community leaders to be knowledgeable about the importance of exclusive breastfeeding within the first six months after childbirth to ensure the healthy growth of young infants. Such an intervention will contribute towards increasing the percentage of exclusive breastfeeding among children under 6 months in Tanzania, which has increased substantially over time, from 26% in 1991–1992 to 64% in 2022 [6] In Myanmar, studies reported that the implementation of community-based interventions aimed at promoting exclusive breastfeeding practices was proven effective [38]. On one hand, some studies recommended promoting the empowerment of women to minimize the existing gender inequalities in accessing and using food and other resources, which impact their ability to maximize their potential. Women empowerment is regarded as instrumental for enhancing infant and young child feeding among other things [39]. On the other hand, other studies have shown a need to empower communities as a whole to enable community members to define and make healthy dietary and lifestyle choices that will help them to improve their overall health status [27]. This study suggests that strategies should be designed to empower both parents collaboratively, fostering a sense of community and shared responsibility. This means that once parents, community leaders and the community in general are all empowered to solve nutritional issues, this will ultimately contribute to the eradication of poor socio-cultural practices that cause undernutrition for under-five children.

Related to community empowerment, this study also suggested that media channels and especially community-based radio programs are an important actor (system) in addressing children’s undernutrition. This needs to be established at the community level in order to convey comprehensive nutrition-based messages including those that would not normally be easily delivered from a distance by healthcare professionals and contribute to dietary behavioral change at the grassroots’ level. Community-based radio programs can also be used to convey key messages to the community during the village Health and Nutrition Days. These events can create competitions for families that perform well in childcaring, and awards can be given to the best family winners as a recognition for their best practices on eliminating child undernutrition. However, it should be noted that the idea of having competitions during the village Health and Nutrition Days has potential limitations including disempowerment. This is due to fact that on one hand, while the findings suggest that introducing competitions could be a motivating factor for families to excel in childcare, on the other hand, competitions may inadvertently lead to a sense of pressure and comparison among families, possibly creating an environment that discourages collaboration and shared learning. Therefore, perhaps the village Health and Nutrition Days should be used more as a platform for disseminating nutritional messages to the community members by nutritionists and influential leaders.

Mass communication has been widely acknowledged as the best channel for effective public health program implementation. Examples of channels include social and digital media such as text messaging, mobile applications, social networking sites, websites and blogs [40]. It was also argued that communication using various channels has contributed largely to increasing community’s awareness and knowledge, modifying their attitudes and behaviors on a multitude of health practices regarding diet and lifestyles [41,42]. However, it should be noted that the recommendation for the use of different communication channels including radio programs is a positive step towards disseminating nutrition-based messages. Nevertheless, the discussion should consider the potential limitations, such as the challenge of the accessibility of radio or other channels of communication in all households and the need for a comprehensive strategy to ensure effective behavior change beyond just message delivery.

In India, it was reported that use of Information, Communication and Education (IEC) is one of the strategies for reinforcing desirable nutrition behaviors among target audiences. Furthermore, entertainment–education (E-E) is another strategy designed to educate the community on nutritional issues, as it seeks to instruct its audience with educational lessons that integrate some forms of entertainment such as radio programs, television (TV) programs, podcasts, games, films and music [43]. However, participatory communication has been reported as an effective approach to inform community members of nutritional issues, since it focuses on how people perceive their food environment, which can help to create a local need and demand for change. Participatory communication involves communities participating in discussions around nutritional issues and solutions with their feedback collected as an evidence base to inform future interventions [44]. Our study findings suggest that whatever channel of communications is used to inform communities about nutritional issues, it should be participatory since it will allow the beneficiaries of the messages to understand the importance of the information communicated to them and thus be able to change their behaviors.

In this study, it was also suggested that at the community level, tackling nutritional issues should become a standing agenda in WDCs since this organ is responsible for overseeing social and economic development issues at the ward level. Making solving nutritional issues a permanent agenda in WDCs will help to build a culture of making close follow-ups on various nutritional interventions that are being implemented at the village level and communicating the progress and outputs timely and regularly to the communities. In addition, the WDC will be answerable to the District Local Government Authorities (LGAs) regarding the implementation of interventions to tackle nutritional issues at the ward level and will make LGAs aware of the progress made in various interventions taking place at the community level. In this case, community-level structures such as wards become an important linking organ with district authorities in overseeing the process of solving nutritional issues. However, an operational challenge was observed in a study conducted on nutrition governance at the sub-national level in Tanzania. It was reported that at the sub-council level, the inclusion of nutrition as a standing agenda in the quarterly ward and village/street development committee meetings had not been well implemented, as the Executive Officers were not made aware of their duties and responsibilities in that agenda, and they did not have adequate capacity and guiding documents for solving nutritional issues [45]. Similar findings were reported in Ogun State, Nigeria, indicating that WDCs were engaged in several activities including health promotion, environmental waste and sanitation education and the promotion of immunization programs at the community level. However, a number of challenges were identified which hindered the effectiveness of such committees in implementing their regular functions. Such limitations included the following: inadequate financial resources and a lack of equipment such as public address systems (PASs) and instructional materials for creating awareness of social and economic development activities at the community level [46]. The findings from the current study suggest that despite the fact that sub-council-level organs such as wards and villages have important roles to play in implementing nutrition-related interventions, capacity-building programs should be established to empower these structures in order to allow them to effectively and efficiently perform their functions.

Given the fact that most of the health-harmful cultural practices are occurring at the community level, the establishment of by-laws aimed at eradicating such cultural practices was recommended as one of the best strategies to tackle child undernutrition. Such by-laws will be protective against the excessive use of alcohol and masculinity practices that accelerate child undernutrition at the community level. In addition, effective village by-laws are expected to support other efforts aimed at ensuring the adherence to WASH practices, eating nutritious foods and economic empowerment for women such as land ownership. Economically independent women will be able to make rational decisions regarding the storing of adequate food at the household level and various nutritional foods for their children. In Malawi, similar findings were reported showing that community leaders play various roles including advising, regulating and restricting cultural practices that promote the violation of adolescents’ rights as well as formulating by-laws for handling sexual abuse complaints in their respective communities [47]. By formulating by-laws, community leaders are playing their major role of providing and ensuring justice in the community. This study suggests that community leaders as regulators and justice providers can assist in bringing much needed change in dealing with child undernutrition. However, it is worth noting that any village by-laws that will be established need careful consideration, ensuring they are culturally sensitive and respectful.

The healthcare delivery system, which is closer to the community level, and particularly health centers and dispensaries were viewed as an instrumental organ for the provision of regular health education on exclusive breastfeeding to pregnant and lactating women and community members, in order to change their behavior towards exclusive breastfeeding practices. In addition, healthcare workers can support the development of innovative ideas such as developing RUTFs based on locally available ingredients. It is the dietitians’ and nutritionists’ responsibility to maintain optimal nutrition for patients and clients. Other studies reported similar findings showing the importance of healthcare workers’ role in increasing patients’ awareness of nutritional issues. For instance, nurses and other healthcare workers have been performing multidisciplinary tasks including assessing the nutritional status of patients due to lacking dietitians and nutritionists at the health facility level [48,49]. A study in Ethiopia reported barriers and facilitators to the implementation of nutritional interventions at primary healthcare units. The main barriers included the lack of functional equipment for anthropometric measurement, poor staff commitment, motivation and the lack of priority for nutrition service, poor knowledge and belief among service providers about the intervention, and the lack of active involvement and support from leaders [50]. Healthcare delivery systems through the facility management team can also link with national-level organs through the submission of regular reports showing the nutritional status of their patients, thus helping national-level organs to take appropriate measures to eradicate the problem of undernutrition among under-fives and other populations in their catchment areas.

District-level systems were also reported in this study as an important actor in the fight against undernutrition for under-fives, especially if they can play their main role of mobilizing and allocating financial resources for implementing nutrition-based activities within councils (Table 2). In so doing, LGAs can create an enabling environment for nutrition professionals to carry out their nutrition-related responsibilities smoothly. This idea was mentioned as a district-level strategy to fight against child undernutrition. However, it was further noted that insufficient resources were allocated to implement nutritional interventions at the district level. Similar findings regarding the role of the district health system were reported in Ghana indicating that the district implements most key nutritional interventions including behavior-change-focused communication on infant and young child feeding, micronutrient supplementation, and growth promotion. However, similar to Tanzania, it was noted that the district health system in Ghana faced several constraints affecting the effective implementation of nutritional interventions. These limitations included the low prioritization in nutrition, inadequate financial commitment, the shortage of healthcare workers and insufficient job aids [51]. Furthermore, it was revealed that insufficient investment in nutritional interventions prevented the delivery of quality nutrition services in the district, and district nutritionists had been shifted to perform other non-nutritional tasks [51]. Similar challenges of implementing nutritional interventions at the lower-level system were identified in Guatemala [52] and Burkina Faso [53]. The studies suggested that the lack of prioritization in nutritional interventions indicated that district health planners perceived nutrition as secondary to disease control. Therefore, having effective and sustained nutritional interventions may translate into improved nutrition among children at the district level.

Furthermore, council authorities can initiate mobile field classes (farmer field schools) to educate community members on nutritionally diversified foods, thereby improving communities’ access to diversified diets. The LGAs can use experts in nutrition and agriculture to conduct educational campaigns to community members about producing and eating healthy foods including fruits and vegetables to help fight undernutrition among under-fives. In addition, it was pointed out that the health and education departments in district authorities should collaborate to strengthen school feeding programs for all primary schools in order to avoid child starvation at schools. The district department of education is responsible for supervising the implementation of school feeding programs in their respective areas. In Nepal, the district-level implemented School Health and Nutrition program (SHN) provided education on nutritional issues to students and surrounding communities. Such a program had contributed to the improvement in students’ health and school environment and enhanced community’s awareness of nutritional issues. However, limited funding and the lack of coordination between stakeholders were the major barriers toward implementing a sustainable SHN program in Nepal [54]. A review of the role of district health management teams in childcare strategies has found that in low- and middle-income countries (LMICs), district management teams are responsible for overseeing all health services provided in their respective areas including child immunization and growth monitoring. However, inadequate resources and the lack of skilled healthcare workers for prioritizing childcare interventions have made the district health system ineffective in the provision of district health services [55].

At the national level, it was suggested that national actors including the Ministry of Education and other stakeholders should put more emphasis on the integration of nutritional knowledge in primary/secondary schools’ curricula with the focus of eliminating the existing health-harmful cultural beliefs and practices that have accelerated the problem of child undernutrition in communities. Furthermore, national-level actors should ensure that the nutritional guidelines developed by the Ministry of Education are implemented in schools so as to improve the nutritional status among school-age children. A systematic review of integrating nutrition into the education sector in LMICs documented similar findings suggesting a need for well-integrated and culturally sensitive nutritional and health education into the existing school curricula. Such education must be taught by skilled personnel from different sectors including those from nutrition and public health and school staff. This finding suggested that children who obtained proper nutritional education at primary and secondary school levels were able to fight malnutrition when they become parents in the future.

It was also recommended that national-level organs such as the Ministry of Health, Agriculture and other government agencies should conduct a regular supervision and close monitoring of the implementation of different interventions on improving the nutritional status of people at regional and district levels, including ensuring that the national nutrition guidelines are implemented at the grassroots’ level (Table 2). Another study conducted in Tanzania reported that national-level organs including ministries of education, agriculture and industries have formulated policies and strategies to combat child undernutrition. Such policies and strategies focus on increasing the purchasing power of the lower-income groups so as to allow poor people to compete more fairly in the market and be able to acquire sufficient food and also on increasing agricultural production by empowering people to control their food production [56]. Similar to any other developing countries, Tanzania needs to recognize the magnitude of child undernutrition’s issue and put in place appropriate strategies, legislations, policies and long-term solutions for tackling such issue [56].

Furthermore, in our study, it has been revealed that national-level organs including the Prime Minister’s Office, President’s Office responsible for Regional Administration and Local Government, Ministry of Health, government agencies and other stakeholders are important actors for creating national forums that will bring together interested individuals from all walks of life to discuss on nutritional issues including strategies of eliminating poor cultural practices that lead to child undernutrition in the country. Bundara et al. [56] reported similar findings showing that it is essential for the government of Tanzania to involve all key stakeholders in areas of community development, education, finance and agriculture to examine how different stakeholders’ policies could help achieve improved childhood nutrition. According to this study, a cross-sector approach has been termed ‘Health in All Policies’ implying multisectoral collaboration, which integrates various efforts from different stakeholders both within and outside health sectors [57,58]. Other studies have also shown the importance of collaboration between the health sector and other sectors [59,60]. The 2020–2023 USAID report on advancing nutrition in Tanzania has shown a close collaboration between the government and international organizations in supporting efforts towards the elimination of malnutrition in the country. Some of the implemented activities include strengthening the Prime Minister’s Office (PMO) and the Tanzania Food and Nutrition Centre (TFNC) through supporting capacity-building sessions and the introduction of planning and budgeting tools. Such efforts have resulted in an increase in the number and quality of nutrition-based activities in the country [60].

The current study findings suggest that in order to achieve the target of eliminating child undernutrition in Tanzania, integrated effective coordination within the nutrition sector and between other sectors at all levels (systems approach) is of paramount importance, as it will facilitate an easy identification of the multiple causes of childhood malnutrition including social–cultural factors, thereby jointly looking for relevant and appropriate solutions to address them. In addition, the effective coordination of all sectors will also ensure the efficient utilization of resources and make all responsible systems accountable for the elimination of child undernutrition in the country.

## 5. Study Limitations and Strengths

This study has three main limitations. The first one is about the selection of nutritionists, medical doctors, nurses and other stakeholders in nutritional health as study participants without including community members who are culture users. This limitation has led to missing the contribution of ideas from community members who are engaged in day-to-day social–cultural practices. Nevertheless, the inclusion of people such as nutritionists and other key stakeholders who have worked and interacted with communities for a long period has helped them build sufficient experience that enabled them to provide valuable insights into culturally-sensitive strategies for the elimination of child undernutrition for under-fives in the community. The second study limitation is about the implementation of the research design, in which the FGDs mixed participants from different regions, instead of conducting FGDs with participants from the same region, which would help the research team to collect the experiential insights of key stakeholders in developing feasible, practical and culturally-sensitive strategies for effectively preventing child undernutrition in the respective regions of Tanzania. However, since the five regions involved in this study are from the same zone of the country (Southern Highlands), we believe that the proposed strategies are feasible, practical and culturally sensitive for effectively preventing child undernutrition in these regions, as they share a more or less similar culture. The third study limitation is about the timing of the study. The study was conducted three years ago (2021), which implies that there may be some possible social, economic and cultural changes that may have occurred in the country for the past three years, which may question the validity of the findings. However, despite such limitations, we still believe that given the study design and triangulation of methods used in this study, the generated findings provided an in-depth understanding of various feasible and culturally-sensitive strategies that can be practically implemented by sectors at different organizational levels (from the community- to national-level systems) for addressing the persistent challenge of undernutrition among under-five children in Tanzania.

## 6. Conclusions

Our findings highlighted that collaboration among various stakeholders from all levels (national to community) is important for the effective planning, coordination and implementation of nutritional interventions aimed at addressing childhood malnutrition. The administrative systems in the nutritional sectors at different levels should prioritize nutritional interventions in their plans, and allocate adequate human and non-human resources to implement culturally-sensitive nutritional interventions towards the elimination of childhood undernutrition. There is a need to strengthen institutional capacity including putting in place legal frameworks, advocacy policies and monitoring mechanisms to ensure that all challenges limiting the implementation of nutritional interventions at the district- and lower- levels are identified in order to sustainably and successfully implement these interventions. All these interventions from the national to community levels require collaborative governance through a systems approach to enable all key stakeholders to play their roles effectively.

## Figures and Tables

**Table 1:** Socio-demographic characteristics of study participants.

Characteristics	National Level (*n* = 20) Number (%)	Regional Level (*n* = 20) Number (%)	Total (*n* = 40) Number (%)
Sex			
Male	9 (45)	7 (35)	16 (40)
Female	11 (55)	13 (65)	24 (60)
Age (years)			
25–34	4 (20)	4 (20)	8 (20)
35–44	9 (45)	11 (55)	20 (50)
45–55	4 (20)	4 (20)	8 (20)
>55	3 (15)	1 (5)	4 (10)
Level of education			
Certificate	3 (15)	3 (15)	6 (15)
Diploma	1 (5)	1 (5)	2 (5)
Bachelor’s degree and above	16 (80)	16 (80)	32 (80)
Cadre			
Medical doctor	2 (10)	2 (10)	4 (10)
Health officer	2 (10)	4 (20)	6 (15)
Nurse	1 (5)	4 (20)	5 (12.5)
Nutritionist	11 (55)	6 (30)	17 (42.5)
Others (administrator, pharmacist)	4 (20)	4 (20)	8 (20)

Source: similar demographic characteristics were presented in our sister paper [21].

**Table 2:** Summary of the systems approach to prevent undernutrition for under-fives in Tanzania.

**Sub-district system level** (ward and village level): Develop culturally sensitive and respectful by-laws to guide communities towards changing their behaviors and adapting good dietary practices and to prioritize and implement sustainable dietary, hygiene and sanitation interventions at the community level. Through healthcare workers at health centers and dispensaries and other extension officers, educate and counsel communities on good and sustainable dietary and hygiene practices to fight against child undernutrition and other vulnerable populations within the communities. Receive feedback from the communities via community health workers and individual people regarding the implementation of nutritional interventions at the communities. Take appropriate measures against those who exercise bad cultural practices that promote child undernutrition, based on the available community by-laws and national legal frameworks. Work with LGAs to implement all identified priorities in nutritional interventions and provide feedback to the district-level system regarding the opportunities and challenges in implementing nutritional interventions at the community level. **District-level system**: Allocate adequate local resources and prioritize and implement culturally sensitive nutritional interventions in district health plans. Supervise and monitor the implementation of various nutritional interventions including safe, affordable and sustainable diets and improved food environments and/or dietary practices in the district. Communicate with higher-level national organs regarding the challenges faced in the implementation of nutritional interventions and suggest possible strategies to be incorporated into national-level policies, guidelines and legal frameworks. **National-level system:** The Ministry of Education, Agriculture, Health and others: The allocation of resources (human and non-human) and the development of guidelines, policies, strategies and legal frameworks to ensure the effective implementation and monitoring of sustainable nutritional interventions at the community level and surrounding environments including schools. National-level systems are also expected to advocate for the implementation of safe and sustainable dietary practices at the community level using policies and strategies and through working with the Local Government Authorities and other implementing partners.

## Data Availability

For privacy and ethical reasons, generated data for this study cannot be shared publicly, however, they are available upon request from the first author.

## Supplementary Materials

The following supporting information can be downloaded at: https://www.mdpi.com/article/10.3390/nu16111551/s1, Figure S1: Southern Highland regions of Tanzania (Rukwa, Njombe, Iringa, Songwe, and Ruvuma regions).
