# Supplementary material for: A Systems Approach in the Prevention of Undernutrition among Children under Five in Tanzania: Perspectives from Key Stakeholders
Source: Nutrients. 2024 May 21;16(11):1551. doi: 10.3390/nu16111551 (PMC11174600; doi:10.3390/nu16111551)
Supplement: Supplementary file 1 [file nutrients-16-01551-s001.zip › nutrients-2952153-supplementary.pdf]

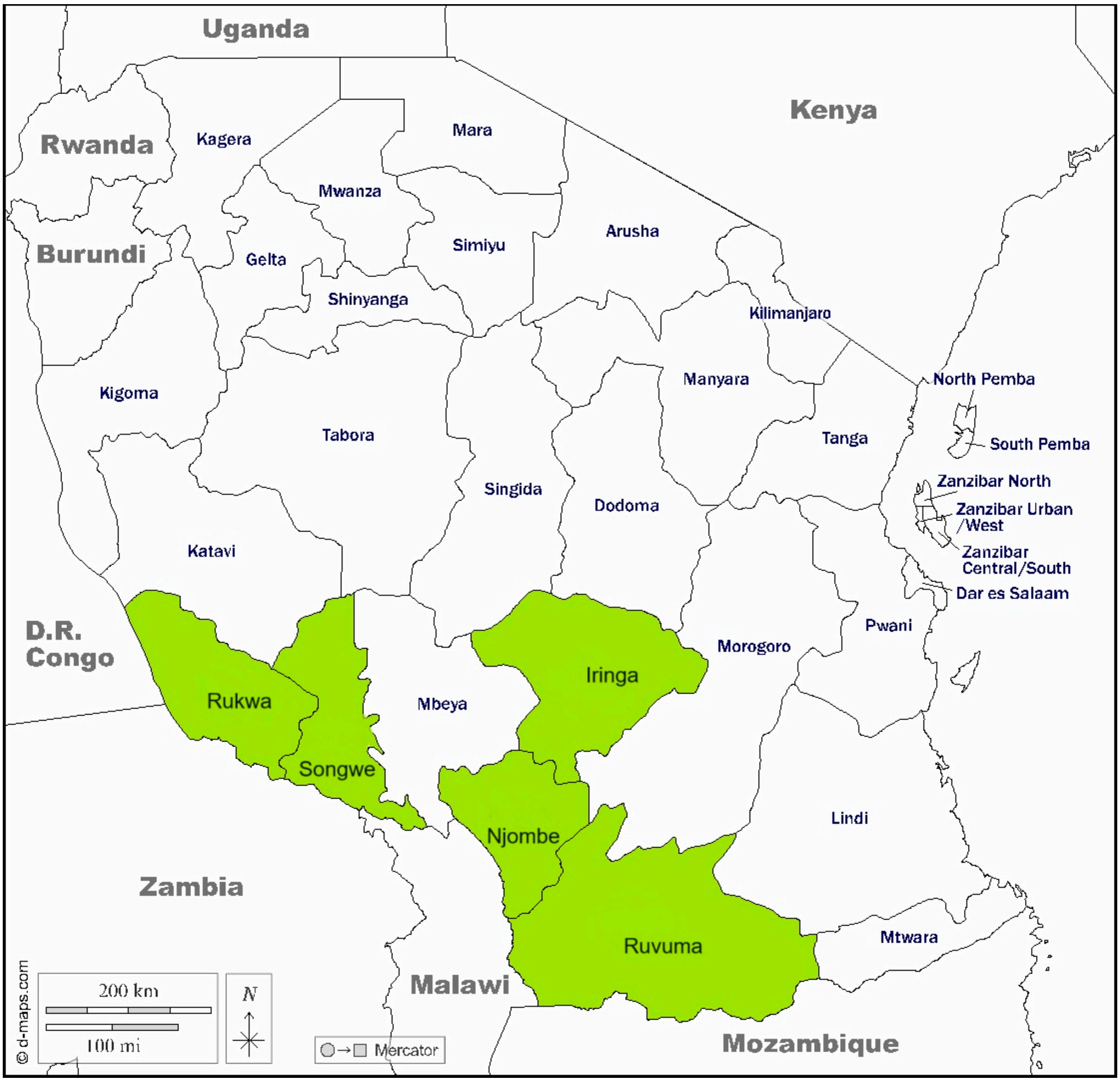

Figure S1. Tanzania Map showing the study area: Southern Highland regions (Rukwa, Njombe, Iringa, Songwe, and Ruvuma regions)
